# Mr. Frederick Victor Milward

**Published:** 1910-04-09

**Authors:** 


The death is announced at Edgbaston, Birmingham, on the 31st ult., of Mr. Frederick Victor Milward, M.B., B.C. (Cantab.), F.R.C.S. (England). Mr. Milward was educated at Cambridge and St. Thomas's Hospital; he was forty years of age, and was surgeon to out-patients at the Children's Hospital, Birmingham; assistant surgeon and surgical tutor at the General Hospital, Birmingham; and surgeon to the Orthopædic and Spinal Hospital in the same city. He also held appointments in the Ambulance Brigade and the First Southern General Hospital of the Territorial Medical Service. He had contributed frequently to professional literature.

